# Wanted dead or alive? Using metabarcoding of environmental DNA and RNA to distinguish living assemblages for biosecurity applications

**DOI:** 10.1371/journal.pone.0187636

**Published:** 2017-11-02

**Authors:** Xavier Pochon, Anastasija Zaiko, Lauren M. Fletcher, Olivier Laroche, Susanna A. Wood

**Affiliations:** 1 Coastal and Freshwater Group, Cawthron Institute, Nelson, New Zealand; 2 Institute of Marine Science, University of Auckland, Auckland, New Zealand; 3 Marine Science and Technology Centre, Klaipeda University, Klaipeda, Lithuania; 4 Environmental Research Institute, University of Waikato, Hamilton, New Zealand; University of Hyogo, JAPAN

## Abstract

High-throughput sequencing metabarcoding studies in marine biosecurity have largely focused on targeting environmental DNA (eDNA). DNA can persist extracellularly in the environment, making discrimination of living organisms difficult. In this study, bilge water samples (i.e., water accumulating on-board a vessel during transit) were collected from 15 small recreational and commercial vessels. eDNA and eRNA molecules were co-extracted and the V4 region of the 18S ribosomal RNA gene targeted for metabarcoding. In total, 62.7% of the Operational Taxonomic Units (OTUs) were identified at least once in the corresponding eDNA and eRNA reads, with 19.5% unique to eDNA and 17.7% to eRNA. There were substantial differences in diversity between molecular compartments; 57% of sequences from eDNA-only OTUs belonged to fungi, likely originating from legacy DNA. In contrast, there was a higher percentage of metazoan (50.2%) and ciliate (31.7%) sequences in the eRNA-only OTUs. Our data suggest that the presence of eRNA-only OTUs could be due to increased cellular activities of some rare taxa that were not identified in the eDNA datasets, unusually high numbers of rRNA transcripts in ciliates, and/or artefacts produced during the reverse transcriptase, PCR and sequencing steps. The proportions of eDNA/eRNA shared and unshared OTUs were highly heterogeneous within individual bilge water samples. Multiple factors including boat type and the activities performed on-board, such as washing of scientific equipment, may play a major role in contributing to this variability. For some marine biosecurity applications analysis, eDNA-only data may be sufficient, however there are an increasing number of instances where distinguishing the living portion of a community is essential. For these circumstances, we suggest only including OTUs that are present in both eDNA and eRNA data. OTUs found only in the eRNA data need to be interpreted with caution until further research provides conclusive evidence for their origin.

## Introduction

The spread of non-indigenous species (NIS) represents a significant and increasing risk to the ecosystem functioning and services of the receiving environment [1,2]. In marine systems, NIS that survive the transport and adapt to new locations can have significant adverse effects on local biodiversity, including the displacement of native species, and shifts in biological communities and associated food webs [3,4]. Severe economic loss attributable to NIS have also been documented in the tourism, aquaculture, and other industry sectors [5–8]. Once NIS are established, they are extremely difficult and costly to eradicate [9,10], and further regional spread may occur through natural dispersal or via anthropogenic transport pathways [10–12]. While vessel hull fouling and ships’ ballast waters are well known as important anthropogenic pathways for the international spread of NIS [1,13–15], comparatively little is known about the potential of regionally transiting vessels to contribute to the secondary spread of marine pests through bilge water translocation.

Recent studies have revealed that the water and associated debris entrained in bilge spaces of small vessels (<20 m) can act as a vector for the spread of NIS at regional scales [16–21]. Bilge water is defined as any water that is retained on a vessel (other than ballast), and that is not deliberately pumped on board. It can accumulate on or below the vessel’s deck (e.g., under floor panels) through a variety of mechanisms, including wave actions, leaks, via the propeller stern glands, and through the loading of items such as diving, fishing, aquaculture or scientific equipment [22]. Bilge water, therefore, may contain seawater as well as living organisms at various life stages, cell debris and contaminants (e.g., oil, dirt, detergent, etc.), all of which are usually discharged using automatic bilge pumps or are self-drained using duckbill valves. Bilge water pumped from small vessels (manually or automatically) is not usually treated prior to discharge to sea, contrasting with larger vessels that are required to separate oil and water using filtration systems, centrifugation, or carbon absorption [22,23]. If propagules are viable through this process, the discharge of bilge water may result in the spread of NIS.

Fletcher et al. [21] used a combination of laboratory- and field-based experiments to investigate the diversity, abundance, and survival of biological material contained in bilge water samples taken from small coastal vessels. Their laboratory-based experiment showed that ascidian (*Ciona* spp., *Didemnum vexillum*) colonies or fragments, and bryozoan (*Bugula neritina*) larvae, can survive passage through an unfiltered pumping system largely unharmed. They also conducted the first morpho-molecular assessment (using eDNA metabarcoding) on the biosecurity risk posed by bilge water discharges from 30 small vessels (sailboats and motorboats) of various origins and sailing time. Using eDNA metabarcoding they characterized approximately three times more taxa than via microscopic methods, including the detection of five species recognized as non-indigenous in the study region.

To assist in understanding the risks associated with different NIS introduction vectors, traditional microscopy-based biodiversity assessments are increasingly being complemented by eDNA metabarcoding (e.g. [24–27]). This allows a wide range of diverse taxonomic assemblages, at many life stages to be identified. It can also enable the detection of NIS that may have been overlooked using traditional methods. Despite the great potential of eDNA metabarcoding tools for broad-scale taxonomic screening [28,29], a key challenge for eDNA in the context of environmental monitoring of marine pests, and particularly when monitoring enclosed environments such as some bilge spaces or ballast tanks, is differentiating dead and viable organisms [30]. Extracellular DNA can persist in dark/cold environments for extended periods of time (months to years [31,32], thus many of the organisms detected using eDNA metabarcoding may have not been viable in the location of sample collection for days or weeks. In contrast, ribonucleic acid (RNA) deteriorates rapidly after cell death, likely providing a more accurate representation of viable communities [33]. Recent metabarcoding studies have explored the use of co-extracted eDNA and eRNA molecules for monitoring benthic sediment samples around marine fish farms and oil drilling sites [34–38], and have collectively found slightly stronger correlations between biological and physico-chemical variables along impact gradients when using eRNA. From a marine biosecurity prospective, the detection of living NIS may represent a more serious and immediate threat than the detection of NIS based purely on a DNA signal. Environmental RNA may therefore offer a useful method for identifying living organisms in samples.

The aim of the present study was to explore the biodiversity patterns of putatively dead and alive taxonomic assemblages contained in bilge water samples previously described in Fletcher et al. [21], using metabarcoding analysis of co-extracted eDNA and eRNA. We hypothesized that the recovery of Operational Taxonomic Units (OTUs) found only in the eDNA group of a bilge water sample represent legacy DNA from dead organisms, whereas OTUs either simultaneously recovered from both eDNA and eRNA (shared) signatures or unique to the eRNA group correspond to living taxa. The specific objectives were; (1) to assess global biodiversity patterns recovered from each studied group (DNA-only, shared, and RNA-only) based on OTU data, (2) to investigate how vessel types (sailboat versus motorboat) influenced the composition of biological assemblages in each group, and (3) to evaluate methodological considerations for future applications of eDNA/eRNA metabarcoding in marine biosecurity.

## Materials and methods

### Bilge sample collection

Fifteen bilge water samples (Table 1) were collected from two different types of small, coastal vessels (yachts and motorboats) between January to March 2015, from marinas in Nelson (41°15.47’S, 173°16.95’E) and Picton (41°17.3’S, 174°0.8’E), New Zealand. Bilge water volumes ranged from 0.2–18.8 L (mean: 6.22 ± 1.69).

**Table 1:** Date of sample collection, sampling location, boat type, volume of bilge, boat use, and port of origin.

Sample No.	Sampling date	Sampling location	Boat type	Volume of bilge water, L	Boat use	Port of origin
1	8-Jan-15	Nelson	Yacht	18.8	Recreational	Brisbane, Australia
2	15-Jan-15	Nelson	Yacht	2.6	Recreational	Auckland, New Zealand
3	17-Jan-15	Nelson	Yacht	1.9	Recreational	Picton, New Zealand
4	26-Jan-15	Nelson	Yacht	0.2	Recreational	Wellington, New Zealand
5	30-Jan-15	Nelson	Motorboat	12.8	Research	Nelson, New Zealand
6	2-Feb-15	Nelson	Yacht	2	Recreational	Dunedin, New Zealand
7*	11-Feb-15	Picton	Motorboat	16.5	Research	Picton, New Zealand
8*	11-Feb-15	Picton	Motorboat	17.6	Research	Picton, New Zealand
9	18-Mar-15	Nelson	Motorboat	3.2	Research	Nelson, New Zealand
10**	23-Mar-15	Nelson	Yacht	2.7	Recreational	Tasmania, Australia
11**	23-Mar-15	Nelson	Yacht	1.7	Recreational	Tasmania, Australia
12	28-Mar-15	Nelson	Motorboat	3.3	Recreational	Nelson, New Zealand
13	28-Mar-15	Nelson	Motorboat	1.7	Recreational	Nelson, New Zealand
14	28-Mar-15	Nelson	Motorboat	5.5	Recreational	Nelson, New Zealand
15	28-Mar-15	Nelson	Motorboat	2.8	Recreational	Nelson, New Zealand

*Samples collected at two different times from the same research vessel and trip.

**Samples collected from two distinct bilge water spaces of the same recreational yacht.

All yachts were sampled within 24 hours of arrival at the marinas. Ports of origin were regional, national and international; however, both international vessels had initially entered New Zealand at Opua near to the northern tip of New Zealand. Bilge water from yachts was sampled directly from bilge water reservoirs (e.g., bilge sump beneath floor panels within the vessel’s cabin) using a sterile hand pump or syringe. Motorboats were generally sampled upon their return to the marina boat ramp following removal from the water (the vessel bung was removed and all entrained water collected). Two of the motorboats sampled were small vessels (<8 m length) used primarily for scientific research purposes; discharges from these vessels were collected separately over the duration of routine trips. Prior to each sampling event, all sampling equipment was thoroughly washed using 2% bleach solution and rinsed with Milli-Q water. After collection, the samples were placed on ice and immediately transported to the laboratory. Triplicate subsamples from each collected sample (30 mL, 45 in total) were filtered using GF/C filter papers (1.6 μm pore size, Whatman International Ltd., Maidstone, UK) and the filters stored at -80°C until DNA/RNA extraction.

### DNA and RNA extractions and high-throughput sequencing

The filters were placed into ZR BashingBead Lysis Tubes (2.0 mm; Zymo Research, CA, USA) containing Lysis Buffer (1 mL) from the ZR-Duet^™^ DNA/RNA MiniPrep Kit (Zymo Research, CA, USA), and placed on a beat beater for 10 mins. DNA and RNA were then co-extracted from filters using the ZR-Duet^™^ DNA/RNA MiniPrep Kit (Zymo Research, CA, USA), following the manufacturer’s protocol. The quality and purity of isolated RNA and DNA were checked on 1.5% agarose gels and using a Nanophotometer (Implen, Munich, Germany). Trace DNA molecules carried over in RNA extracts were eliminated by two sequential DNase treatments as in Langlet et al. [39]. The efficiency of the DNase treatment was verified by running a 50-cycle PCR analysis on all RNA samples using the reagents (e.g., DNA primers) and conditions used for the down-stream 18S rRNA amplification (see below). This PCR verification yielded no amplified products, indicating complete elimination of DNA traces in these samples. Extracted RNA was reverse transcribed using the SuperScript^®^ III reverse transcriptase (Life Technologies, CA, USA). The various extract products (DNA, cDNA and RNA) were separated into aliquots and stored frozen (-20°C for DNA/cDNA and -80°C for RNA) until further analysis. For all DNA (n = 15) and corresponding co-extracted cDNA (n = 15) samples, hereafter referred to as eDNA and eRNA, an Illumina MiSeq^™^ library was generated following a two-step tailed PCR amplicon procedure [40]. The universal primers Uni18SF and Uni18SR [41] were used to amplify the eukaryotic V4 region of the nuclear small subunit ribosomal DNA (18S rRNA) gene. The primers were modified to include Illumina^™^ overhang adaptors as described in Kozich et al. [42]. PCR amplifications (n = 30) were undertaken on an Eppendorf Mastercycler (Eppendorf, Germany) in a total volume of 20 μL using AmpliTaq Gold^®^ 360 PCR Master Mix (Life Technologies), 2 μL GC enhancer, 0.8 μL of each primer (IDT DNA, CA, USA) and 1–2 μL of template eDNA/eRNA. Reaction cycling conditions were: 95°C for 3 min, followed by 32 cycles of 94°C for 30 s, 50°C for 30 s, 72°C for 90 s, and a final extension of 72°C for 7 min. To ensure amplification of uncontaminated products, all PCR included negative controls (no template samples). No contamination was detected in any instance.

Amplicons were purified using the AMPure^™^ XP system (Agencourt, USA) and quantified using the Qubit BR dsDNA kit (Invitrogen, USA), and diluted to a concentration of 1 ng/μL. Library preparation and HTS was conducted at New Zealand Genomics Limited (NZGL), University of Auckland. Sequencing adapters and sample-specific indices were added to each amplicon via a second round of PCR using the Nextera^™^ Index kit (Illumina^™^). In order to assess the robustness of sequencing and analytical pipeline, internal sequencing quality (positive) DNA controls were applied as described in Zaiko et al. [25].

Amplicons were pooled into a single library and paired-end sequences (2 × 250 base-pairs) generated on a MiSeq instrument using the TruSeq^™^ SBS kit (Illumina^™^). Sequence data were automatically demultiplexed using MiSeq Reporter (v2), and forward and reverse reads assigned to samples.

### Bioinformatics analyses

Bioinformatics analysis of metabarcoding data was performed using VSEARCH tool [43]. All reads resulting from the HTS run were assessed for quality, and any read that contained a base with the reported Phred quality score below 30 was discarded. Forward and reverse paired-end sequences were assembled independently for each sample. Merged reads that were less than 200 base-pairs in length were discarded. The data were then filtered, discarding all reads that had more than one expected error per read [44].

Sequences within each triplicate were then pooled (separately for eDNA and eRNA). This resulted in a set of 15 paired samples, which were then rarefied down to the lowest sequence number in each pair for further downstream analysis.

The retained sequences were de-replicated into unique sequences and clustered at 97% identity threshold. Reads were then mapped against the representative set of sequences generated in the clustering step and taxonomy was assigned against the Protist Ribosomal 2 (PR2) database [45] at 97% threshold, using the UCLUST assigner implemented in QIIME [46]. In order to reduce the potential introduction of artefact sequences [47], OTUs represented by fewer than 10 sequences across the entire dataset were discarded. The taxonomic assignments were verified against the World Register of Marine Species, AlgaeBase, Encyclopedia of Life and Integrated Taxonomic Information System databases. Sequences corresponding to organisms of terrestrial origin were intentionally kept in the datasets as they may be representative of legacy DNA from non-living biodiversity. Quality filtered eDNA and eRNA sequence data, OTU and taxonomy tables are provided in the Supporting information (S1 File).

### Statistical analyses

Rarefaction curves for each sample were generated using the vegan package [48] implemented in the R v3 statistical computing environment [49]. Venn diagrams for visualizing the relation of OTUs composition of eDNA and eRNA origin, were generated using the VennDiagram package [50] implemented in the R v3.

A pairwise comparison of relative abundance of OTUs (percentage of sequence reads per OTU) of eDNA and eRNA origin from each sample was performed using Wilcoxon signed-rank test with continuity correction implemented in R. The Bonferroni α-correction was applied for multiple pairwise tests. The D3 JavaScript library (https://d3js.org/) was used for visualising the taxonomic composition from metabarcoding data.

Non-metric multi-dimensional scaling (nMDS) analyses, undertaken using Jaccard similarity matrices and implemented in the PRIMER 7 statistical software [51], were used to visualize in two-dimensional space the partitioning of variation in; *i*) eDNA and eRNA OTU composition (presence-absence) between yachts and motorboats, and *ii*) taxonomic composition (taxonomic composition aggregated at Phylum-level) between eDNA-only, eDNA/eRNA-shared, and eRNA-only biodiversity on yachts and motorboats.

Similarity Percentages analysis (SIMPER [52]) implemented in PRIMER 7 was used to identify the percentage contribution of taxa (genus level) to observed pairwise differences between eDNA and eRNA samples.

## Results

### High-throughput sequencing

The stringent filtering and subsampling of the raw sequencing data resulted in 1,670,539 high-quality sequence reads with 3,173 OTUs generated. A total of 1,437 OTUs were represented by 10 or more sequences per dataset and were therefore retained for downstream analyses. Rarefaction curves (S1 Fig), indicated that most eDNA and eRNA samples (combined triplicate subsamples) were adequately sequenced, except for the eRNA sample #13 where the rarefaction curve did not reach a plateau. This sample and the corresponding eDNA sample were removed from further analyses. As described in Fletcher et al. [21], the internal positive DNA control samples yielded 131,068 high-quality sequence reads, clustered into 10 OTUs. Of those, 3 OTUs (99.7% of sequences) were assigned to the target taxa at expected relative abundances.

**Fig 1:**
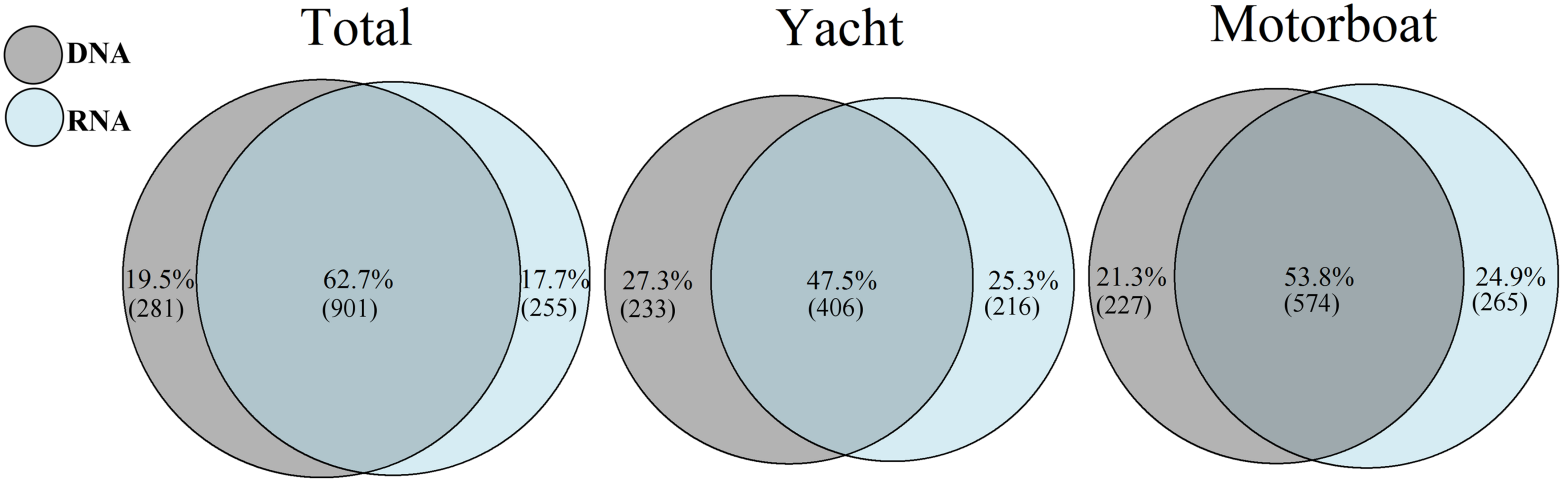
Venn diagrams showing the percentage of DNA-only, shared eDNA/eRNA and RNA-only Operational Taxonomic Units (OTUs) in all samples, as well as in samples from yachts and motorboats. Numbers in brackets correspond to the number of OTUs in each group.

Most of the OTUs (78%; represented by 1,556,611 sequences) were identified as eukaryotic organisms, with more than 99% of them (1,556,284 sequences) assigned to phylum or lower taxonomic levels (S1 Table). Of the classified OTUs from the complete sequence dataset, 62.7% (1,461,709 sequences) were present in both eDNA and eRNA datasets (‘shared OTUs’), the rest were found exclusively in the eDNA (19.5% OTUs, 68,850 sequences; ‘eDNA-only OTUs’) or eRNA (17.7% OTUs, 25,725 sequences; ‘eRNA-only OTUs’; Fig 1). The ratio of shared OTUs did not differ markedly between yacht and motorboat samples (47.5% and 53.8%, respectively), as well as proportion of eDNA-only (27.3% and 21.3%) and eRNA-only OTUs (25.3% and 24.9%, Fig 1).

### Taxonomic diversity: eDNA versus eRNA

The Wilcoxon signed-rank test showed no statistically significant pairwise differences in relative total abundance of OTUs between eDNA and eRNA datasets (p = 0.29). However, when pooling samples by boat type, the pairwise relative total abundance of OTUs in motorboat samples differed significantly between eDNA and eRNA (p<0.001), while in samples collected from yachts the difference remained insignificant (p = 0.11).

**Fig 2:**
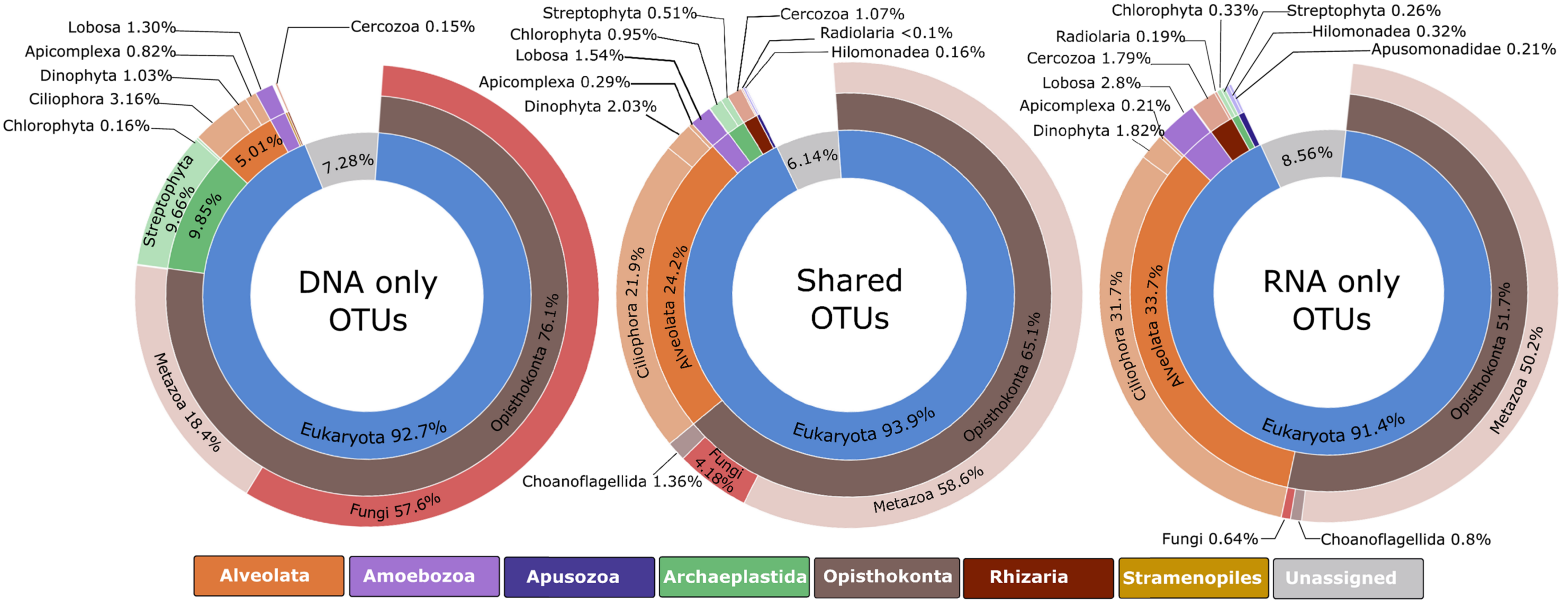
Global biodiversity of Operational Taxonomic Units (OTUs) for the DNA-only, shared eDNA/eRNA, and RNA-only datasets. The charts show the relative abundance of sequences at highest assigned taxonomic levels.

Fig 2 shows the relative proportions of taxonomic composition obtained from total abundance of sequences per OTU among the following three datasets: ‘eDNA-only’ (281 OTUs), ‘shared eDNA/eRNA’ (899 OTUs), and ‘eRNA-only’ (255 OTUs).

The most abundant taxa in the eDNA-only OTUs were fungi (57.6% of sequences), followed by metazoans (18.4%) and streptophytes (9.6%), while the shared eDNA/eRNA and eRNA-only OTUs were consistently dominated by metazoans (50.2–55.2%) and ciliates (20.6–31.7%; Fig 2). A marked increase (11.1%) in ciliate sequences was observed from the shared eDNA/eRNA OTUs to the eRNA-only OTUs.

**Fig 3:**
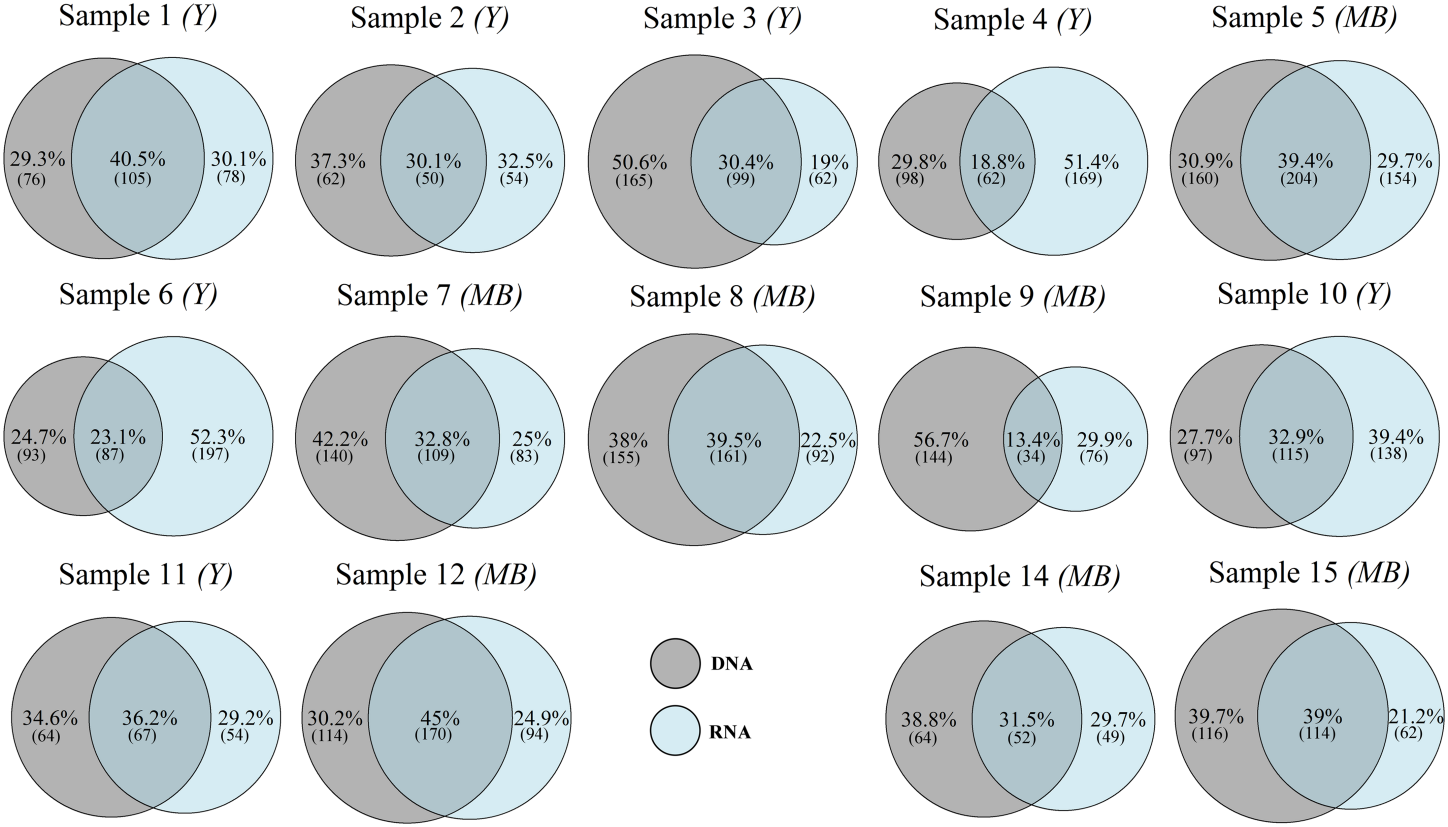
Venn diagrams showing the percentage of DNA-only, shared eDNA/eRNA, and RNA-only Operational Taxonomic Units (OTUs) in individual pairs of eDNA and eRNA samples. Numbers in brackets correspond to the number of OTUs in each group. Samples from either yachts (*Y*) or motorboats (*MB*) are indicated.

The pairwise analysis of OTU composition of eDNA and eRNA reads revealed that the proportion of shared OTUs within individual samples varied between 13 to 45%, with on average 37% of eDNA-only OTUs and 31% of eRNA-only OTUs (Fig 3).

The Wilcoxon signed-rank test showed that the abundance of OTUs differed significantly between corresponding eDNA and eRNA datasets in seven samples (Table 2).

**Table 2:** Results of the pairwise comparison of relative abundance (percentage of sequence reads) of Operational Taxonomic Units (OTUs) between eDNA and eRNA datasets in each sample. Wilcoxon signed-rank test P-values are indicated, with significant values shown in bold.

*Sample No*.	*Boat type*	*No. of OTUs*	*Wilcoxon test P-value*
*1*	Yacht	259	0.29
*2*	Yacht	166	0.03
***3***	**Yacht**	**326**	**<0.001**
*4*	Yacht	329	0.23
***5***	**Motorboat**	**518**	**<0.001**
***6***	**Yacht**	**377**	**<0.001**
*7*	Motorboat	332	0.15
***8***	**Motorboat**	**408**	**<0.001**
*9*	Motorboat	254	0.59
***10***	**Yacht**	**349**	**<0.001**
*11*	Yacht	184	0.65
*12*	Motorboat	378	0.6
***14***	**Motorboat**	**155**	**<0.001**
***15***	**Motorboat**	**292**	**<0.001**

In most cases, the taxa that drove these differences had higher abundances of eDNA sequences (S2 Fig). However, there were a few exceptions, with more abundant eRNA sequences in some samples. For example, among protists, a free-living amoeba *Vermamoeba*, ciliates *Aristerosoma*, *Favella* and *Suctoria* spp., and an heterotrophic dinoflagellate *Oxyrrhis*; and among multicellular organisms; nematodes, copepods, hydrozoans, and a bony fish *Auxis* spp.

**Fig 4:**
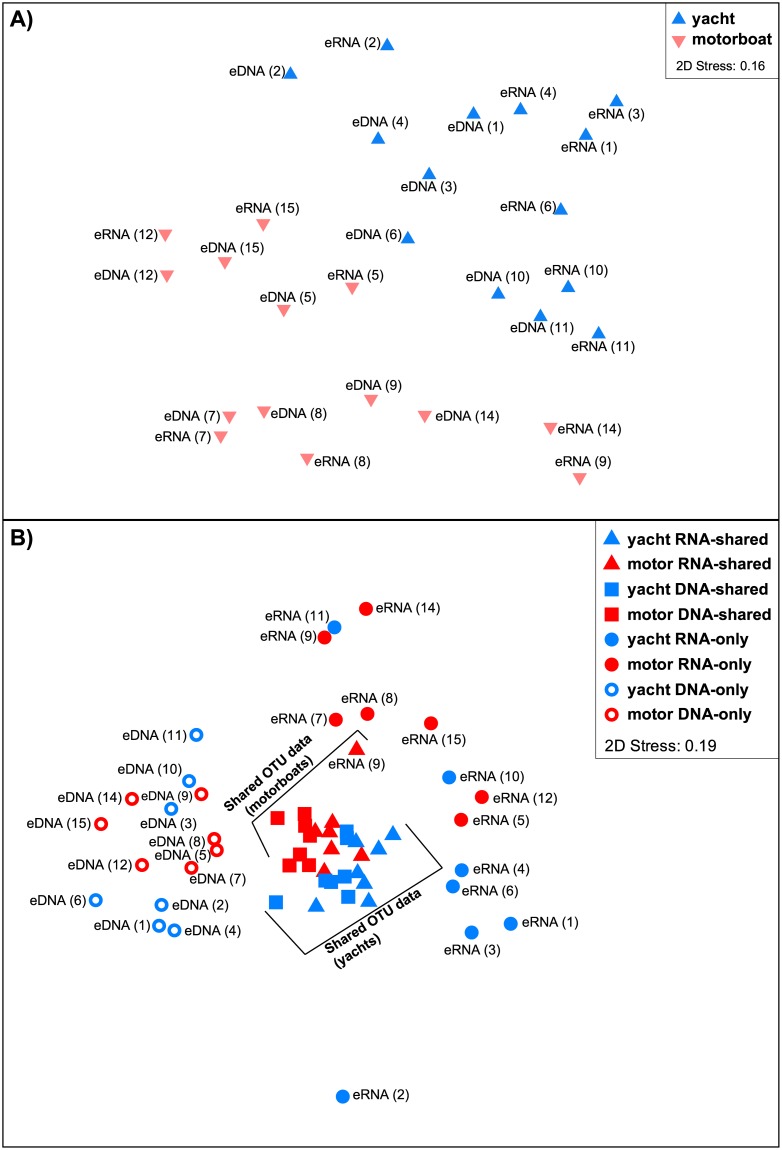
Non-metric multi-dimensional scaling (nMDS) plot analyses. Plots are constructed using Jaccard similarity matrices from; (**A**) presence-absence of Operational Taxonomic Units (OTU)-based diversity between the global eDNA and eRNA datasets collected on yachts versus motorboats, and (**B**) taxonomic composition (presence-absence) based on OTU data aggregated at Phylum-level, split into eDNA-only, eDNA/eRNA-shared, and eRNA-only for samples from yachts and motorboats. Sample numbers are indicated in parentheses (refer to Table 1).

The nMDS analysis plot based on presence-absence of OTU-based diversity between global eDNA and eRNA datasets from bilge water samples collected from yachts and motorboats (Fig 4A), showed that there was a clear separation in community composition between vessel types. Additionally, the community composition of OTUs isolated within vessel types (i.e., yachts versus motorboats) from eDNA and eRNA extracts was, in general, mostly similar between identical samples, with a few exceptions such as samples 3, 4, 6, and 9.

The nMDS analysis derived from the presence-absence of eDNA-only, eDNA-shared, eRNA-shared, and eRNA-only OTUs, aggregated at the phylum-level from yacht versus motorboat datasets, showed a very contrasting result (Fig 4B). While the eDNA/eRNA-shared data yielded one closely aggregated cluster (visually separated by vessel types), the eDNA-only and eRNA-only data yielded two markedly separated and more diffuse clusters with no obvious separation by vessel types.

## Discussion

### High heterogeneity in co-extracted eDNA and eRNA molecules in bilge water samples

Over the last five years, eDNA metabarcoding [53,54] has emerged as a novel monitoring method for a variety of applications, including biodiversity estimates and invasive species detection [24,26,41,55,56]. The strong structural integrity of DNA molecules enables their persistence in the environment (known as environmental DNA) for extended periods of time following cell death [31,32]. In aquatic environments this has provided opportunities for large scale detection of a wide range of species, including those present only at low densities or which are difficult to identify using traditional methods [57]. However, because of the persistence of DNA in the environment, eDNA metabarcoding results are of limited use for inferring living biodiversity. This may be problematic in some situations such as measuring the success of applied treatments, eradication or control programmes, where determining the presence of living organisms is essential [30,58]. Environmental RNA is known to degrade within minutes to hours [58–61], and therefore is expected to provide a better proxy for characterizing living organisms. Several studies have used eRNA and suggest that in most cases it is more effective than eDNA for characterizing metabolically active species [34,37,38,62,63].

The present study explored diversity patterns of OTUs recovered from co-extracted eDNA and eRNA molecules isolated from bilge water samples of small (<20 m) motorboats and yachts traveling regionally. Similar to previous studies [34,36,64,65], our results showed that a larger proportion of the OTUs were found in both the eDNA and eRNA reads. There were also a considerable proportion of OTUs exclusively found in either the eDNA-only or eRNA-only reads. While the recovery of OTUs found only in the eDNA reads can be explained through the detection of DNA from dead organisms as well as extracellular DNA (free-floating or legacy DNA) that has bound to surrounding particles [66], the recovery of eRNA-only molecules is more difficult to justify.

At the global dataset scale (Fig 1), the majority of OTUs were identified at least once in both eDNA and eRNA (shared) reads with just under 20% of OTUs unique to either eDNA or eRNA datasets only. However, striking differences in the taxonomic diversity were observed between the eDNA-only, eRNA-only and shared groups (Fig 2). For example, over 57% of the eDNA-only OTUs corresponded to fungi sequences. Bilge water environments experience drastic fluctuations in water temperature, dryness and sun exposure, salinity, and contaminant concentrations, all of which may influence the survivorship and accumulation of resistant organisms [21]. Fungi are able to thrive in a wide range of extreme conditions, including dry and cold habitats [67], highly alkaline sites [68], and environment with high Ultra-Violet rays [69,70]. The most likely explanation for the high proportion of fungal OTUs found exclusively in the eDNA group, is that they represent legacy DNA from dead fungi that have accumulated through time in the vessel’s bilge spaces. This could have resulted in a bias or enhanced amplification of fungal 18S rRNA signatures compared to other organisms during the PCR stages.

In contrast, the shared eDNA/eRNA OTUs were dominated by metazoan (55.2%) and ciliate (20.6%) sequences, with only a small fraction (4.8%) of fungal sequences. Similar proportions were observed in the eRNA-only group, although there was a marked increase in ciliates (31.7% sequence reads). One possible explanation for the high proportion of ciliate sequences in the eRNA-only group, is that they are the result of increased cellular activity combined with unusually complex genome organization. Gong et al. [71] recently reported that ciliates generally have much higher rDNA copy numbers than other protists and fungi (up to 310,000 rDNA copies per cell), which could lead to overestimation of the relative abundance of ciliates in environmental samples when rDNA sequence-based methodologies are used. Gong et al. [71] further argued that although there are numerous copies of rDNA in ciliate macronucleus, it is likely that only a small portion of these genes are transcriptionally active. Our results suggest that this may not be the case, and that a higher rDNA copy number in actively living ciliate communities may translate into enhanced transcription rates and transcript products which are preferentially picked up via eRNA metabarcoding. Additional research is required to test this hypothesis. A further possibility is that some rare taxa might be amplified and identified in the eRNA due to lower abundance of other taxa (i.e., those responsible for the accumulation of legacy DNA). This could be further enhanced if these taxa have increased cellular activity.

Another potential explanation is that a considerable portion of OTUs in the eRNA samples are artefacts. Laroche et al. [65] summarized the range of PCR artefacts potentially occurring during RNA preparation steps. As cited from the latter study, these may include: *i*) the incorporation of point mutations in some of the cDNA sequences by non-proof reading reverse transcriptase [72,73]; *ii)* the jumping of transcriptase from one template to another (template-switching), which may produce either chimeric cDNA sequences from intermolecular template switching or shortened isoform sequences from intramolecular template switching [74]; *iii*) the introduction of nucleotide biases at the beginning of the 5’-end of sequences originating from the use of random hexamers primers during cDNA synthesis [75], and *iv)* other PCR and sequencing errors [76]. Laroche et al. [65] highlighted that the use of RNA controls (e.g., synthetic oligomers) and technical (PCR) replicates could help identifying these artefacts and improve concordance between the eDNA and eRNA profiles.

**Fig 5:**
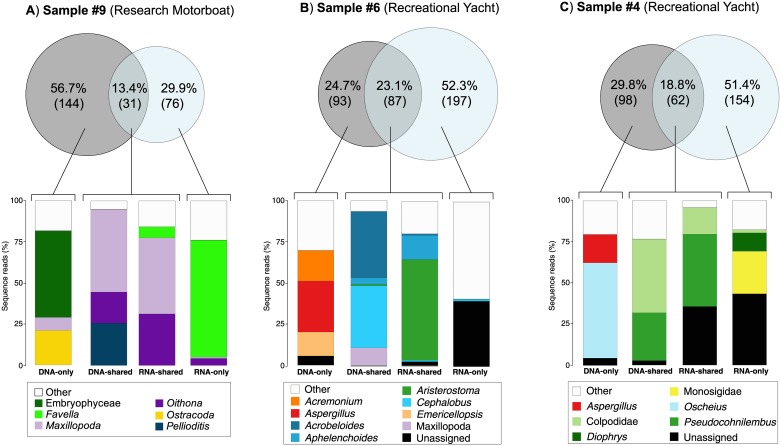
Sequence reads proportions of the <10 most abundant Operational Taxonomic Units (OTUs) in the eDNA-only, eDNA-shared, eRNA-shared, and eRNA-only portions of (A) sample 9, (B) sample 6, and (C) sample 4.

The relative proportions of eDNA-only and eRNA-only OTUs recovered within each individual bilge water sample were highly heterogeneous (Fig 3). While some samples showed a consistent distribution among the three groups with approximately 40% of shared OTUs and between 20–30% of OTUs restricted to either eDNA-only or eRNA-only (e.g., samples 1, 11, and 12), other samples contained far greater portions of either eRNA-only or eDNA-only OTUs (e.g., samples 4, 6, and 9; Fig 5).

The latter three examples may each highlight the methodological scenarios mentioned above. Sample 9 had a high portion (56.7%) of eDNA-only OTU’s (Fig 5A). This sample was from a research vessel where washing down scientific equipment on the deck is a regular practice. This would result in a high number of dead organisms (i.e., legacy DNA) entering the bilge system and likely explains the high portion of eDNA-only OTUs represented by plants (Embryophyceae), crustaceans (Ostracoda), and copepods (Maxillopoda). In sample 6 (Fig 5B), the eDNA-only OTUs were largely composed of legacy DNA of fungi (*Aspergillus* spp., *Acremonium* spp., and *Emericellopsis* spp.), contrasting with the eRNA-shared OTUs, which were dominated by ciliate (*Aristerostoma* spp.) sequences, possibly aligning with the scenario of high cellular activity and transcription rates in ciliates. Among the eRNA-only OTUs in sample 6, there were 41 ‘other’ distantly related taxa (data not shown). This may represent an example of the detection of ‘rare’ taxa, which were either not detected in the more complex eDNA samples or which have increased cellular activities thereby enhancing their detection. In sample 4, 51.4% of the OTUs were only found in the eRNA dataset. The diversity analysis (Fig 5C) showed that while eDNA-only OTUs were dominated by nematodes (*Oscheius* spp.) and fungal (*Aspergillus* spp.) sequences, the eRNA-only group contained a high number of unassigned sequences, perhaps corresponding to living taxa that have not yet been genetically described and which are absent from available sequence reference databases. Although the bioinformatics pipeline used in the present study included stringent quality filtering and chimera removal, we cannot exclude the possibility that at least some of these unassigned sequences are the result of PCR artefacts potentially generated during cDNA library preparation (see more detailed discussion above). Despite increasing attempts to develop protocols to reduce these artefacts (e.g. [77]), further research is required to fully understanding these possibilities.

### Effect of vessel type on biological diversity

The global OTU-based nMDS analysis showed a clear separation between community structure in the samples collected from motorboats and yachts (Fig 4A). In this study, the yacht operators reported that water mostly entered the vessels from waves, minor leaks or cooling of the propeller shaft. Fletcher et al. [21] suggested that this water would most likely have been sourced from offshore locations. In contrast, motorboats operators indicated that the origin of the bilge water was primarily associated with sporting and wash-down activities. Since these activities take place relatively close to the shore, Fletcher et al. [21] proposed that the source of the water is the most likely explanation for the observed differences in community structure between vessel types. The nMDS analysis of eDNA-only, eDNA/eRNA-shared, and eRNA-only OTUs diversity at the phylum-level (Fig 4B) provided further insights into the potential origin of eRNA-only OTUs. This analysis demonstrated marked separation between eDNA-only, eDNA/eRNA-shared, and eRNA-only assemblages, indicating a pronounced taxonomic divergence suggesting that, as discussed above, the eRNA OTUs could be caused by artefacts during PCR or reverse transcription analyses, and rare taxa not identified in the more complex eDNA samples, or due to an over-expression of their rRNA transcripts.

### Challenges and promises of using eRNA in marine biosecurity

For the purpose of marine biosecurity surveillance, an indication of presence of an unwanted organism would often trigger a tiered management response (e.g., [78], which may involve visual surveys, and further sampling for molecular and morphological assessments. In this case, eDNA signal from biodiversity screening would be sufficient to launch targeted detection and rapid response actions. However, there are examples where information on whether organisms within a sample are living is required, for example, monitoring ballast water to control compliance with the International Ballast Water Management Convention [79,80], acquiring approval for ballast water treatment systems [81], or for determining the success of a control or eradication programme. A variety of techniques have been used to determine whether organisms are alive including: visual counting, culturing, motility assessments, vital staining, flow cytometry, fluorometry, and immunoassays [82,83]. These methods all have limitations and are often protracted. For example, many organisms cannot be cultured, motility assays involve microscopic observations, which are laborious and only applies to motile taxa, and while florescent staining works well for bacteria and some algae [84,85], it is not suitable for organisms >50 μm in size. In the present study, we demonstrate the utility of eRNA metabarcoding as a method for determining the presence of living organisms within a sample, corroborating previous findings [34,38,86,87]. The relatively short persistence of eRNA is the primary characteristics that makes it suitable for differentiating living and dead taxa, however the susceptibility of RNA to relatively rapid degradation also makes it challenging to work with. Specialized collection and storage protocols are needed (e.g., samples need to be frozen immediately or stored in often expensive preservation buffers), dedicated instruments and sample preparation rooms are required for RNA isolation, and the reverse transcription step adds considerable expense and time to the sample processing. Despite these challenges, and with the on-going advancements in sequencing technologies [88], we advocate that eRNA has significant potential for differentiating the living and dead portions of complex communities in environmental samples, and is a technique that can be up-scaled relatively easily allowing a large number of samples to be analyzed.

## Conclusion

In this study, we explored the diversity of eukaryotic OTUs in bilge water samples from small marine vessels using metabarcoding of co-extracted DNA and RNA. Our results showed that when global data are combined, over 62% of OTUs are recovered at least once in the shared eDNA/eRNA data, with a considerable proportion restricted to the eDNA- (19.5%) or eRNA-only (17.7%) data. We provide evidence that the eDNA-only OTUs are largely composed of legacy DNA from dead organisms or dormant cells and spores, in particular fungi. Explanation for the presence of OTU in the eRNA-only data are more uncertain and include: *i)* many of the OTUs were from ciliates which are thought to have high rRNA copies which might be preferentially amplified during the PCR, *ii*) the OTUs might be from rare taxa not detected in the eDNA due to the more diverse eDNA communities or due to some taxa more actively expressing rRNA transcripts as a result of increased cellular activity, and/or *iii*) they might include artefacts generated during the reverse transcription and library preparation steps. For generalized marine biosecurity applications (e.g., untargeted surveys or biodiversity screenings), we recommend that all OTUs should initially be examined. Even the presence of an OTU from an NIS in the eDNA-only group (i.e., legacy DNA) may provide useful information on operating vectors and pathways, or assist in early detection. Where knowledge on the living taxa is required, analyses should focus on the shared eDNA and eRNA OTUs. We suggest that OTUs within the eRNA-only group should also be examined as the detection of rare taxa may be enhanced in the eRNA in some situations. However, signals from eRNA-only OTUs should be interpreted with caution until knowledge on their origin is enhanced. Further research is recommended to improve understanding on the persistence of RNA in the environment, and the underlying reasons for the presence of RNA-only OTUs in environmental samples.

## Supporting information

S1 File Zip file containing quality filtered sequence data (concatenated fasta file), as well as the Operational Taxonomic Units (OTUs) table and corresponding taxonomic table obtained from the bioinformatics pipeline. (ZIP)

S1 Table List of the 1,437 Operational Taxonomic Units (OTUs) retained for downstream analyses, with their assignment at the lowest possible taxonomic level and the number of corresponding sequences recovered for each OTU within the 15 eDNA and 15 eRNA samples investigated. **Note**: Some invasive species listed (e.g., *Septifer biolcularis*, *Mytilus trossulus*) have not yet been recorded in New Zealand. It is possible that these species are present in New Zealand but cryptic, or alternatively that they are the result of assignment mistakes due to the limited resolution of the marker used and/or the lack of comprehensive reference sequence database available for New Zealand. Therefore, without some other form of validation, the species-level assignations should be treated cautiously.
(XLSX)

S1 Fig Rarefaction curves of eukaryotic 18S rRNA sequences collected from the combined bilge water triplicate subsamples from vessels in the Nelson and Picton marinas between January to March 2015, with (A) DNA, and (B) RNA. (TIFF)

S2 Fig Relative abundance of selected taxa (genus level) contributing >10% to pairwise difference in relative abundance of sequences in the DNA and RNA samples (based on SIMPER analysis results). See Table 1 for sample information.
(PNG)
