# Supplementary figures and images for: Wanted dead or alive? Using metabarcoding of environmental DNA and RNA to distinguish living assemblages for biosecurity applications
Source: PLoS One. 2017 Nov 2;12(11):e0187636. doi: 10.1371/journal.pone.0187636 (PMC5667844; doi:10.1371/journal.pone.0187636)

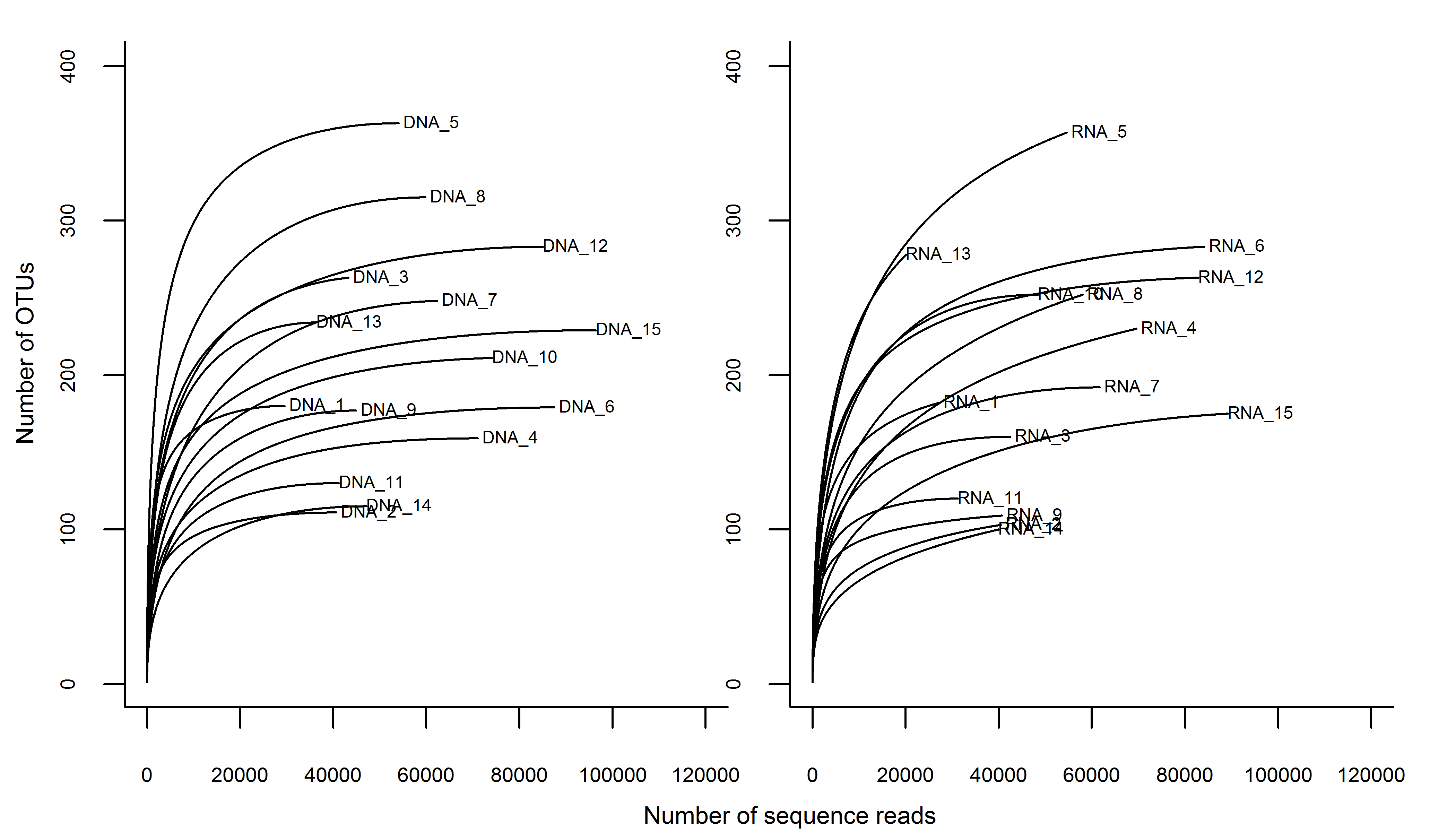

Supplement: S1 Fig — (TIFF) [file pone.0187636.s003.tiff]

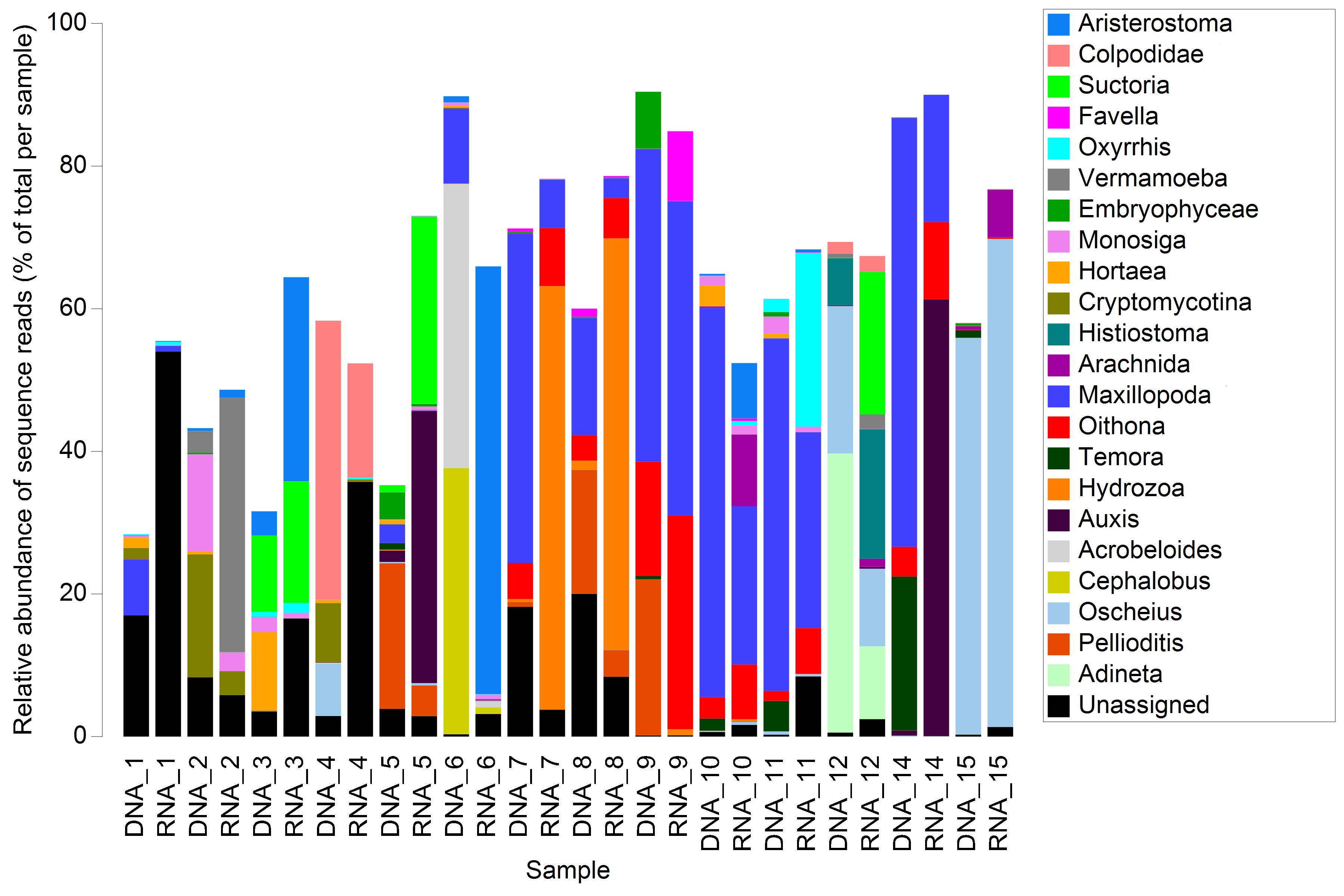

Supplement: S2 Fig — See Table 1 for sample information. (PNG) [file pone.0187636.s004.png]
